# The Risk Factors for Perioperative Serum Albumin Variation in Pediatric Patients Undergoing Major Gastroenterology Surgery

**DOI:** 10.3389/fsurg.2020.627174

**Published:** 2021-01-25

**Authors:** Qingshuang Liu, Kai Gao, Chao Zheng, Chunbao Guo

**Affiliations:** ^1^Department of Pediatric General and Neonatal Surgery, Children's Hospital, Chongqing Medical University, Chongqing, China; ^2^Ministry of Education Key Laboratory of Child Development and Disorders, Chongqing Key Laboratory of Pediatrics, National Clinical Research Center for Child Health and Disorders, China International Science and Technology Cooperation Base of Child Development and Critical Disorders, Chongqing Engineering Research Center of Stem Cell Therapy, Children's Hospital of Chongqing Medical University, Chongqing, China; ^3^Department of Orthopedics, Children's Hospital, Chongqing Medical University, Chongqing, China

**Keywords:** ALB, Roux-en-Y hepaticojejunostomy, postoperative recovery, perioperative complications, pediatric patients

## Abstract

**Background:** The albumin, a negative acute-phase protein, is important for perioperative morbidity, even in patients with normal preoperative levels. This study intend to determine the perioperative factors related with the postoperative reduction in serum albumin (ΔALB) and its influence on perioperative outcome in a pediatric general surgical cohort.

**Methods:** This single-center retrospective review included 939 pediatric patients who underwent major gastroenterology surgery from August 2010 to August 2019. The patients were dichotomized into a high ΔALB group (≥14.6%) and a low ΔALB group (<14.6%) based on the mean value of ΔALB (14.6%). the independent risk factors for ΔALB, were explored using the propensity score matching to minimize potential selection bias and subjected to method multivariable logistic regression model. Furthermore, in 366 matched patients, the influences of operating time on perioperative outcomes were analyzed.

**Results:** Among the 996 patients reviewed, 939 patient records were enrolled in the final analysis. Controlling for other factors, multivariable analysis showed that a high CRP on POD 3 or 4 [odds ratio (OR) = 2.36 (95% CI, 1.51–3.86); *p* = 0.007], a longer operating time [OR = 1.18 (95% CI, 1.00–1.53); *p* = 0.014), and the presence of Charcot's triad [OR = 1.73 (95% CI, 1.05–2.83); *p* = 0.031] were factors that predicted a high ΔALB level. A high ΔALB level was also related with gastrointestinal functional recovery delay, reflected by the postoperative defecation (*p* = 0.013) and bowel movement (*p* = 0.019) delay and the high occurrence of postoperative complications (16.1 vs. 10.9%, OR, 1.57; 95% CI, 1.02–2.41, *P* = 0.0026).

**Conclusions:** The high ΔALB level was correlated with postoperative outcome. To obtain a safe recovery and discharge after a major abdominal operation, the above risk factors for ΔALB could be addressed in the perioperative period.

## Background

As an acute-phase protein, albumin (ALB) usually negatively responds to surgical stress, injury or sepsis (1, 2). A decrease in plasma albumin concentration is considered one of the features of systemic inflammation due to the loss of albumin to the tissue spaces (3, 4) and postoperative infectious complications (4). The transcapillary leakage of albumin can be elevated by more than 300% in systemic inflammatory pathogenesis (5, 6). Preoperative hypoalbuminemia has also been confirmed as an indicator for poor nutritional status, and postoperative complication after spine surgery (7), GI surgery (8), and acute kidney injury (9). Because albumin has quick kinetics after surgery, which can even occur earlier than that of CRP, making it an intense focus of perioperative management (10, 11).

A sharp decrease of 33% in serum ALB within 2 days has been observed after major abdominal surgery (2, 12). There are many factors that may potentially impact the reduction in ALB level (ΔALB), including the pathology being treated and the actual procedure undertaken (13). Few studies have examined the factors that could be used to predict postoperative hypoalbuminemia in pediatric patients after major gastroenterology surgery. A clear evaluation of these factors could lead to the development of optimized perioperative care protocols.

We intended to explore the risk factors for the reduction in ALB by retrospectively reviewing data on pediatric patients that had undergone major gastroenterological surgery. We further sought to clarify the association between ΔALB and postoperative recovery and outcomes.

## Methods

### Population Selection

This retrospective study included 996 consecutive patients managed with elective Roux-en-Y hepaticojejunostomy from August 12, 2010 to August 28, 2019 at our institute. Exclusion criteria included patients with preoperative or postoperative day (POD 1) ALB administration or subjects with incomplete laboratory data. The study protocol was approved by the Ethics Committee of Chongqing Medical University.

### Data Collection and Definitions

Electronic medical records generated upon admission or referral were individually reviewed by 2-well-trained clinical investigators who collected the relevant data. Data extraction included (1) preoperative data, including demographic data and clinical details, such as preoperative neutrophils, lymphocytes, hemoglobin, preoperative CRP and ALB, and pre-existing comorbidities; (2) intraoperative variables, including surgical procedures (surgical approach, type of resection) and (3) postoperative outcomes, gastrointestinal function recovery features and postoperative complications. The involved laboratory data were usually collected preoperatively and on PODs 3 and 7. Gastrointestinal function recovery were evaluated within the postoperative days 5, including the postoperative flatus or defecation, time to normal diet, etc. The postoperative complications were ranked following the Clavien-Dindo classification system (14), such as intra-abdominal abscesses, postoperative hemorrhage, anastomotic fistula, anastomotic stenosis, etc.

The relative serum albumin change (ΔALB) was calculated as (preoperative albumin-nadir albumin level within POD 2)/preoperative albumin × 100% (15). We evaluated ΔALB as normally distributed data and decided to use the mean value of the (14.6%) to dichotomize the groups. The patients were divided with a low (<14.6%) and a high (≥14.6%) ΔALB group based on the cutoff value (14.6%).

The primary outcome based on ΔALB was prompt postoperative gastrointestinal function recovery. The secondary outcomes were postoperative complications and immunologic and inflammatory variables. Gastrointestinal symptoms, like first bowel movement (gas and feces), vomiting, abdominal bloating, abdominal cramps, were assessed and recorded daily for within the postoperative days 5.

### Propensity Scores and Matching and Statistical Analysis

The propensity score matching was firstly performed to minimize selection biases using SPSS 20.0 (IBM, Armonk, NY) or R 3.1.2 (The R Foundation for Statistical Computing). The selected variables entered into the propensity model were demographic data, laboratory values, treatment protocols, surgical features, etc. The assumption of linearity of the PS model was checked using the generalized additive model, subsequently matching 366 patients with high ΔALB ≥ 14.6% and 366 patients with ΔALB <14.6%. The perioperative outcomes between the two groups were compared after propensity score matching.

To verify independent predictors for postoperative complications, multivariate analysis was performed using multivariate logistic regression analysis after univariate analysis to identify predictors with a significance level of *P* < 0.30 (**Table 3**). The results of the multivariate logistic regression analysis were expressed using the *P*-value, odds ratio (OR), and 95% confidence interval (CI). To assess the postoperative outcomes, categorical and continuous variables were analyzed using Fisher's exact test or Pearson's χ2 test as well as Student's *t*-test or Mann-Whitney U test and the Wilcoxon rank-sum test, as appropriate. In all cases, *P* < 0.05 was considered significant.

## Results

### Patient Population

For the whole 996 pediatric patients underwent hepaticojejunostomy resection in our department, 41 did not fulfill the inclusion criteria and were initially excluded, and sixteen patients were excluded because their notes were unobtainable for data extraction. Finally, a total of 939 patient records were enrolled in the final analysis (Table 1).

**Table 1:** Univariate analyses of perioperative factors associated with ΔALB.

	**Total Population**		
	**ΔALB ≥ 14.6%(471)**	**ΔALB < 14.6%(468)**	***p*-values**
Age (yrs), mean ± SD	2.13 ± 1.02	2.16 ± 1.07	0.21^*^
Female: Male	183 (37.5)	191 (39.9)	0.31*$$*
Weight (kg), mean ± SD	11.38 ± 2.96	11.52 ± 3.68	0.22^**^
BMI, median (range)	24 (20–30)	25 (21–31)	0.13^***^
**Laboratory findings**			
Hypertransaminasemia, *n* (%)	325 (69.0)	334 (71.4)	0.47*$$*
Hyperbilirubinemia, *n* (%)	186 (39.5)	173 (37.0)	0.39*$$*
Preoperative ALB (g/L), mean ± SD	39.28 ± 4.72	41.41 ± 5.39	0.016^*^
Preoperative CRP (g/L), mean ± SD	11.24 ± 3.26	12.56 ± 4.17	0.15^*^
Preoperative WBC (109/L), mean ± SD	7.9 ± 2.8	8.2 ± 3.1	0.52^*^
**Ultrasound presentation**			
Mean CBD(cm), mean ± SD	1.53 ± 0.58	2.34 ± 0.97	0.042^**^
Charcot's triad, *n* (%)	142 (30.1)	109 (23.3)	0.011*$$*
Nadir ALB within POD 2 (g/L), mean ± SD	34.94 ± 8.92	30.22 ± 7.83	0.0012^*^
CRP on POD 3 or 4 (mg/L), mean ± SD	41.45 ± 13.65	29.8 ± 7.69	< 0.001^*^
Postoperative WBC (109/L), mean ± SD	15.8 ± 4.9	14.9 ± 4.8	0.350^*^
**Mode of surgical approach**, ***n*** **(%)**			
Laparoscopic	168 (35.7)	139 (29.7)	
Open	303 (64.3)	329 (70.3)	0.03*$$*
Operation time, median (range), min	175 (132–418)	143 (115–367)	0.0024^***^
Nadir of hemoglobin (g/L)	9.18 ± 1.32	9.64 ± 1.68	0.28^*^
Operative blood loss (mL), mean ± SD	36.78 ± 16.84	33.86 ± 15.88	0.26^*^
Intraoperative fluid utilization (mL/kg*h), mean ± SD	16.74 ± 6.88	14.26 ± 6.72	0.017^*^
Intraoperative transfusion, *n* (%)	131 (27.8)	115 (24.6)	0.15*$$*
**ASA classification**			
ASA1-2	338 (72.6)	319 (67.7)	0.13*$$*
ASA3-4	133 (27.4)	149 (22.3)	

*ALB, albumin; ΔALB, reduction of ALB level; ASA, American Society of Anesthesiology; CBD, Common bile duct; CRP, C-reactive protein; POD, postoperative day; SD, standard deviation; WBC, white blood cell*.

Statistical methods:

* Student's t-test;

** Mann-Whitney U test;

*** Wilcoxon rank-sum test;

$$ *Pearson's χ2 test*.

### Factors Associated With ΔALB

The baseline features of the ΔALB ≥ 14.6% and ΔALB <14.6% groups are summarized in Table 1. A larger choledochal cyst size (*p* = 0.042), worse comorbidity (Charcot's triad) (*p* = 0.011), lower preoperative ALB (*p* = 0.016), CRP on POD 3 or 4 (*p* < 0.001), and a longer surgery duration (*p* = 0.0024) were associated with a greater ΔALB (*P* < 0.05) in the univariable analysis. Multivariable analysis revealed three independent risk factors (Table 2) associated with ΔALB, including, CRP on POD 3 or 4 [OR = 2.36 (95% CI, 1.51–3.86); *p* = 0.007], the presence of Charcot's triad [OR = 1.73 (95% CI, 1.05–2.83); *p* = 0.031], and a longer operating time [OR = 1.18 (95% CI, 1.00–1.53); *p* = 0.014].

**Table 2:** Multivariate analysis of perioperative factors associated with ΔALB.

	**OR**	**95%CI**	***P***
BMI > 26	1.26	(0.97–1.89)	0.16
Preoperative ALB < 32 g/L	1.68	(0.91–2.35)	0.27
Preoperative CRP > 12 g/L	1.12	(0.94–1.73)	0.18
CRP on POD 3 or 4 (>135 mg/L)	2.36	(1.51–3.86)	0.007
Charcot's triad	1.73	(1.05–2.83)	0.031
Operation time(>165 m)	1.18	(1.01–1.53)	0.014
Open surgical approach	2.31	(0.89–4.76)	0.31
Intraoperative fluid utilization > 17 mL/kg*h	1.07	(0.96–1.78)	0.22
Intraoperative transfusion	1.38	(0.98–2.16)	0.092
ASA classification(ASA1-2)	1.74	(0.96–3.24)	0.39

*Statistical methods: multivariate logistic regression analysis*.

### Influences of ΔALB on Postoperative Outcomes

To explore the association between ΔALB and perioperative outcome, we performed PS matching between the ΔALB ≥ 14.6% and ΔALB < 14.6% groups. Under PS matching, 366 patients in the ΔALB ≥ 14.6% group were matched to 366 patients in the ΔALB < 14.6% group (Table 3).

**Table 3:** The inclusion variables in the PS-matching analysis.

	**Total Population**		
	**ΔALB ≥ 14.6%(366)**	**ΔALB < 14.6%(366)**	***p*-values**
Age (yrs)	2.14 ± 1.01	2.15 ± 1.03	0.45^*^
Female: Male	135 (36.9)	136 (37.2)	0.50$$
Weight (kg)	11.44 ± 2.76	11.46 ± 3.11	0.34^*^
BMI, median (range)	25 (21–28)	25 (21–29)	0.28^***^
**Laboratory findings**			
hypertransaminasemia, *n* (%)	252 (68.9)	251 (68.6)	0.50$$
hyperbilirubinemia, *n* (%)	146 (39.9)	143 (39.1)	0.44$$
Preoperative ALB (g/L), mean ± SD	39.83 ± 4.26	40.63 ± 4.96	0.18^*^
Preoperative CRP (g/L), mean ± SD	11.87 ± 3.16	12.04 ± 3.88	0.35^*^
Preoperative WBC (109/L), mean ± SD	8.01 ± 2.62	8.14 ± 2.69	0.39^*^
**Ultrasound presentation**			
Mean CBD (cm), mean ± SD	1.75 ± 0.53	2.07 ± 0.86	0.18*^*^
Nadir ALB within POD 2 (g/L), mean ± SD	33.13 ± 8.56	32.34 ± 7.69	0.23^*^
Postoperative WBC (109/L), mean ± SD	15.56 ± 4.64	15.12 ± 4.63	0.46^*^
**Mode of surgical approach**, ***n*** **(%)**			
Laparoscopic	131 (35.8)	126 (34.4)	
Open	235 (64.2)	240 (65.6)	0.38$$
Nadir of hemoglobin (g/L)	9.35 ± 1.25	9.48 ± 1.52	0.33^*^
Operative blood loss (mL)	35.16 ± 14.69	34.47 ± 13.53	0.29^*^
Intraoperative fluid utilization (mL/kg*h), mean ± SD	15.59 ± 6.54	14.93 ± 6.18	0.27^*^
Intraoperative transfusion, *n* (%)	102 (27.9)	98 (26.8)	0.40$$
**ASA classification**			
ASA1-2	262 (71.6)	257 (70.2)	0.37$$
ASA3-4	104 (28.4)	109 (29.8)	

Statistical methods:

* Student's t-test;

** Mann-Whitney U test;

*** Wilcoxon rank-sum test;

$$ *Pearson's χ2 test*.

In the propensity matched cohort, patients with ΔALB < 14.6% had reduced time for postoperative flatus (*p* = 0.013) and postoperative bowel movement (*p* = 0.019) (Table 4). In the ΔALB < 14.6% group, 40.2% (147/366) of patients passed stool within 72 h, and 33.6% (123/366) of patients with high ΔALB passed stool (OR, 1.33; 95% CI, 0.98–1.79, *p* = 0.039). The incidences of diarrhea (*p* = 0.50), vomiting (*p* = 0.38) and abdominal distention (*p* = 0.18) within 5 PODs were similar between the two groups.

**Table 4:** Outcome characteristics in the matched population depended on the mean value of ΔALB.

	**ΔALB > 14.6%(366)**	**ΔALB < 14.6%(366)**	***p*-values**	**Odds ratio (95% CI)**
Hypotensive events, n (%)	42 (10.47)	38 (11.88)	0.36^*^	
Norepinephrine usage, *n* (%)	45 (13.95)	40 (12.17)	0.32$$	
Furosemidum, *n* (%)	36 (9.88)	34 (11.30)	0.50$$	
Metabolic acidosis, *n* (%)	18 (3.20)	13 (4.93)	0.23$$	
Hypokalemic episodes, *n* (%)	24	25	0.50$$	
**Serum albumin**				
First defecation (days)	3.13 ± 1.32	2.88 ± 1.27	0.12^*^	
First flatus	3.56 ± 0.88	3.07 ± 0.90	0.013^*^	
First bowel movement (days), Mean ± SD	2.75 ± 0.82	2.14 ± 0.78	0.019^**^	
Stool within 72 h, *n* (%)	123 (32.75)	147 (40.12)	0.039$$	1.33 (0.98–1.79)
Abdominal distension, *n* (%)	36 (13.5)	28 (19.8)	0.18$$	
Diarrhea, *n* (%)	23 (6.8)	21 (10.9)	0.50$$	
Vomiting, *n* (%)	35 (13.0)	31 (16.1)	0.38$$	
No. of patients with mild complications, n(%)	45 (11.7)	29 (9.0)	0.033$$	1.63 (1.00–2.66)
No. of patients with major complications, *n* (%)	28 (7.7)	21	0.19	
Total number of complications, *n* (%)	59 (16.1)	40 (10.9)	0.026	1.57 (1.02–2.41)
Length of stay (*d*), mean ± SD	8.19 ± 3.16	7.69 ± 2.67	0.057	

Statistical methods:

* Student's t-test;

** Mann-Whitney U test;

$$ *Pearson's χ2 test*.

As shown in Table 4, more total complications, like anastomotic leakage, intraperitoneal abscess, surgical site infections, were found in patients with ΔALB ≥ 14.6% than those with ΔALB < 14.0% (16.1 vs. 10.9%, OR, 1.57; 95% CI, 1.02–2.41, *P* = 0.0026). In addition, the postoperative stay was 8.19 ± 3.16 days in the ΔALB ≥ 14.0% group, which was longer than that of the ΔALB < 14.0% group (7.69 ± 2.67 days), although no statistically significant difference was observed (*p* = 0.057).

## Discussion

We conducted the present analysis to focus on serum albumin as an acute phase protein for the pediatric patients managed with major gastroenterology surgery. This study revealed that several factors were associated with high ΔALB, such as operative duration, disease comorbidities, and mean CBD. Furthermore, a greater decrease in serum albumin were associated with delay in gastrointestinal function recovery, more complications, and prolonged postoperative hospital stay.

The factors leading to hypoalbuminemia are often complicated and associated with operative case type, ALB loss, redistribution, catabolism, or theirs combination (2, 15–17). Numerous researches have focused on hypoalbuminemia as risk factors for postoperative complications (7, 18), while few have specifically stressed the perioperative factors related to ΔALB, which may be clinically significant for postoperative care. In the present study, several clinical factors related to the decrease in postoperative albumin were presented, including longer operating time, severe comorbidities, such as Charcot's triad, and high CRP. During surgery, manipulation of the intestine has been proven to initiate gastrointestinal edema (19, 20). The surgical stress response and postoperative edema might be attributed to the low colloid osmotic pressure and fluid accumulation, themselves resulting from a low postoperative ΔALB (2, 5), which was consistent with the current findings.

The plasma albumin concentration was associated with altered distributions between the intravascular and extravascular space and plasma volume changes. The reasons for this association may pertain to hemodilution, which might contribute to the decreased albumin level after surgery. In our previous study (21), postoperative complications were shown to be related to conventional intraoperative and postoperative fluid usage, which might also affect the albumin level after surgery. Excessive fluid could also promote capillary leakage of serum albumin, which is common in some surgical trauma comdition (6, 22). Serum albumin on POD 3 has been observed to be correlated with preoperative CRP level (13, 23, 24). In the current study, we indeed found that a high ΔALB was associated with CRP on POD 3 or 4.

As indicated in the current study, although uneventful recovery was present for most patients following choledochal cyst resection, unfavorable postoperative gastroenterological recovery was associated with a high ΔALB. Previous biological investigations have suggested that edema should delay the intestinal function recovery through directly affecting muscle function (25, 26). Serum ALB should account for postoperative intestinal edema after a major operation or severe trauma. Furthermore, fluid accumulation could decrease tissue oxygenation, which is also unfavorable for postoperative recovery and complications.

In the current research, we found the trend for complications increase in patients with high ΔALB, including anastomotic leakage. A possible explanation at the tissue level may be that the low ALB level reduced the tissue connections with collagen deposition. The effect of ΔALB on the local inflammatory response and edema recovery is also important and might also explain postoperative recovery and complications (27, 28). It remains to be determined whether ALB supplementation reduces postoperative intestinal edema and cellular swelling in the pediatric population managed with major gastrointestinal surgery (29, 30).

In this research, several weakness should be considered during interpretation the main finding. First, the retrospective design may contribute to selection and treatment biases. Second, there might have been many practice changes over a long period of time in the single center study, which may be different from the current treatment algorithms. Another point of emphasis was that although baseline characteristics were similar after PS matching, the patients with high ΔALB might be surgically more difficult than those with low ΔALB. More solid, multicenter prospective studies should be conducted with less confounding variables to determine the current conclusions.

## Conclusion

In this research, we characterized some risk factors that may predispose patients to high ΔALB, which could negatively impact postoperative recovery after a major abdominal operation in a pediatric population. The surgeons should be aware of ΔALB in the early postoperative period to optimize the surgical care.

## Data Availability Statement

The original contributions generated for the study are included in the article/supplementary material, further inquiries can be directed to the corresponding author/s.

## Ethics Statement

The studies involving human participants were reviewed and approved by the Ethics Committee of Chongqing Medical University. Written informed consent to participate in this study was provided by the participants' legal guardian/next of kin. Written informed consent was obtained from the individual(s), and minor(s)' legal guardian/next of kin, for the publication of any potentially identifiable images or data included in this article.

## Author Contributions

QL and KG designed and analyzed the data. CZ evaluated the manuscript and performed the statistical measurements. CG analyzed the data and wrote the paper. All authors contributed to the article and approved the submitted version.

## Conflict of Interest

The authors declare that the research was conducted in the absence of any commercial or financial relationships that could be construed as a potential conflict of interest.

## Abbreviations

ALB: albumin
ALP: alkaline phosphatase
ALT: alanine aminotransferase
ΔALB: reduction in ALB level
ASA: American Society of Anesthesiology
AST: aspartate aminotransferase
BUN: blood urea nitrogen
CBD: Common bile duct
CI: confidence interval
CRP: C-reactive protein
γ-GTP: gamma-glutamyl transpeptidase
Hb: hemoglobin
LDH: lactate dehydrogenase
OR: odds ratio
POD: postoperative day
Scr: serum creatinine
SD: standard deviation
WBC: white blood cell.
